# The association between physical activity and mental health during the first year of the COVID-19 pandemic: a systematic review

**DOI:** 10.1186/s12889-022-12590-6

**Published:** 2022-02-01

**Authors:** Priscila Marconcin, André O. Werneck, Miguel Peralta, Andreas Ihle, Élvio R. Gouveia, Gerson Ferrari, Hugo Sarmento, Adilson Marques

**Affiliations:** 1 KinesioLab, Research Unit in Human Movement Analysis, Insituto Piaget, Almada, Portugal; 2 CIPER, Faculdade de Motricidade Humana, Universidade de Lisboa, Lisbon, Portugal; 3 Center for Epidemiological Research in Nutrition and Health, Department of Nutrition, School of Public Health, São Paulo, Brazil; 4 ISAMB, Universidade de Lisboa, Lisbon, Portugal; 5 Center for the Interdisciplinary Study of Gerontology and Vulnerability, University of Geneva, Geneva, Switzerland; 6 Swiss National Centre of Competence in Research LIVES – Overcoming vulnerability: Life course perspectives, Lausanne and Geneva, Switzerland; 7 Department of Psychology, University of Geneva, Geneva, Switzerland; 8 Universidade da Madeira, Funchal, Portugal; 9 Interactive Technologies Institute, LARSyS, Funchal, Portugal; 10 Laboratorio de Rendimiento Humano, Grupo de Estudio en Educación, Actividad Física y Salud (GEEAFyS), Universidad Católica del Maule, Talca, Chile; 11 University of Coimbra, Research Unit for Sport and Physical Activity (CIDAF). Faculty of Sport Sciences and Physical Education, Coimbra, Portugal

**Keywords:** Pandemic, SARS CoV-2, Exercise, Mental health, Anxiety

## Abstract

**Introduction:**

The Coronavirus disease-19 (COVID-19) pandemic affected countries worldwide and has changed peoples’ lives. A reduction in physical activity and increased mental health problems were observed, mainly in the first year of the COVID-19 pandemic. Thus, this systematic review aims to examine the association between physical activity and mental health during the first year of the COVID-19 pandemic.

**Methods:**

In July 2021, a search was applied to PubMed, Scopus, and Web of Science. Eligibility criteria included cross-sectional, prospective, and longitudinal study designs and studies published in English; outcomes included physical activity and mental health (e.g., depressive symptoms, anxiety, positive and negative effects, well-being).

**Results:**

Thirty-one studies were included in this review. Overall, the studies suggested that higher physical activity is associated with higher well-being, quality of life as well as lower depressive symptoms, anxiety, and stress, independently of age. There was no consensus for the optimal physical activity level for mitigating negative mental symptoms, neither for the frequency nor for the type of physical activity. Women were more vulnerable to mental health changes and men were more susceptive to physical activity changes.

**Conclusion:**

Physical activity has been a good and effective choice to mitigate the negative effects of the COVID-19 pandemic on mental health during the first year of the COVID-19 pandemic. Public health policies should alert for possibilities to increase physical activity during the stay-at-home order in many countries worldwide.

## Background

The severe acute respiratory syndrome coronavirus-2 (SARS-CoV-2) is a highly contagious virus that infects humans and causes coronavirus disease-19 (COVID-19), which is currently having a damaging impact on almost all countries worldwide [1]. To bring this pandemic to an end, a large share of the world needs to be immune to the virus, and the safest way to achieve this is with a vaccine. Fortunately, in December 2020 the vaccination started in the United Kingdom [2] and is currently pursued in different countries [3]. Until November 2021, 53.3% of the world population has received at least one dose of a COVID-19 vaccine [4]. However, the number of infected people and deaths continues to grow [5]. The World Health Organization (WHO) published a weekly report and on 16th of November 2021 it was observed a increasing trend in new global weekly cases [6]. From the beginning of the pandemic, as a community mitigation strategy used to reduce the spread of COVID-19, most countries adopted the stay-at-home order as well as the stimulation of facemask wearing and hygiene habits [7, 8].

As a consequence of the stay-at-home strategies, mainly during the first year of the COVID-19 pandemic, studies had reported multiple behaviour changes. Some common impacts include disturbed eating behaviours [9], changes in alcohol consumption [10], and substance use [11]. A wide range of psychological outcomes has been observed during the virus outbreak, including a reduction in well-being as well as increases in depressive and anxiety symptoms [12, 13]. Considering the need for social distancing measures, the investigation of possible factors that can mitigate the negative effects of social distancing on mental health should help the promotion of intervention strategies.

Physical activity (PA) is well recognised as a key factor for the prevention and management of mental illness, including mental disorders such as depression and anxiety as well as the promotion of mental health such as well-being [14, 15]. Nevertheless, globally, approximately 23% of adults and 81% of adolescents do not meet the WHO recommendations regarding PA for maintaining health [16–18]. This situation even worsened with the COVID-19 pandemic. Studies have demonstrated that PA declined and sedentary behaviour increased during the COVID-19 pandemic stay at home order in many countries, regardless of the subpopulation [19]. Different studies sought to investigate whether these changes in PA were associated with mental health indicators during the COVID-19 pandemic and a previous systematic review synthetised that PA is an effective strategy to face the psychological effects of the COVID-19 pandemic [20]. However, the previous review included articles published between 1 January 2019, and 15 July 2020, before the second wave of the COVID-19 pandemic. Therefore, our systematic review aimed to update those findings and clarify if PA is associated with mental health during the first full year of the COVID-19 pandemic and to analyse if PA mitigates the effects of the stay-at-home order on mental health. We aimed to explore the first year of the COVID-19 pandemic because it was the period when restrictive orders were strictest when people were strongly encouraged to comply with the stay-at-home order.

## Methods

### Design

This systematic review focuses on peer-reviewed journal articles on the relationship of PA to mental health during the COVID-19 pandemic published until 30 July 2021.

### Data sources and searches

A systematic review protocol was registered with the PROSPERO database on the 29th of January 2021 (IDCRD42021233921). A broad search strategy was employed. Searches were conducted on the 30th of January 2021, in the following electronic databases: PubMed, Scopus, and Web of Science. The search was performed in the three databases using the terms: (physical activity OR physical inactivity OR exercise OR training OR sport* OR fitness OR physical function* OR movement behavio* OR sedentary behavio*) AND (mental health OR psychological health OR depress* OR anxiety OR psychological function* OR mental function* OR wellbeing OR well-being OR burnout OR burn-out OR fear OR fears OR worries OR worry) AND (coronavirus disease OR COVID-19 OR SARS-CoV-2 OR lockdown OR shutdown OR quarantine OR confinement OR social isolation). These terms were searched in title and abstract of scientific articles. Additionally, cross-referencing search was performed in the full-text read of potentially included articles.

### Study selection

Observational studies (cross-sectional, prospective, or longitudinal) were eligible for this review. Furthermore, studies were also required to meet the following criteria: (1) assessing PA by a validated instrument, (2) assessing mental health by a validated instrument, (3) presenting an analysis on the association between PA and mental health. Studies with samples including pregnant women, chronic disease patients, athletes, COVID-19 survivors, and frail older adults were excluded. Besides, studies reporting PA as a moderate or mediated variable were also excluded. Two co-authors screened titles and abstracts to identify articles that met the inclusion criteria. Two co-authors read the articles and decided whether they should be included in the analysis or excluded. The inclusion decision was consensual and in cases of disagreement, the decision was made by mutual agreement.

### Data extraction and synthesis of results

Data extraction was completed independently by one co-author. Data extracted from all studies included study details (author, year of publication, study design, recruitment processes, and date and location of the study); participant characteristics (sex, mean age); outcome and instruments, and main findings. A table was made for articles that analysed the association between PA and mental health among adults, and another table for the analyses of the association between PA and mental health among children and adolescents.

### Quality assessment

The risk of bias was assessed by two independent reviewers, using the Newcastle-Ottawa Scale (NOS) [21] which was also adapted for cross-sectional studies [22]. Therefore, we used the original scale for cohort studies and the adapted scale for the cross-sectional studies. The original scale varies between 0 and 9, while the adapted scale for cross-sectional studies varies between 0 and 10, with higher scores indicating research of better quality.

### Narrative synthesis

Considering the heterogeneity of methods used for the estimation of the exposures and outcomes, it was not possible to conduct a meta-analysis. Therefore, we compared the findings across the included articles according to each outcome.

## Results

### Results of the search

**Fig. 1:**
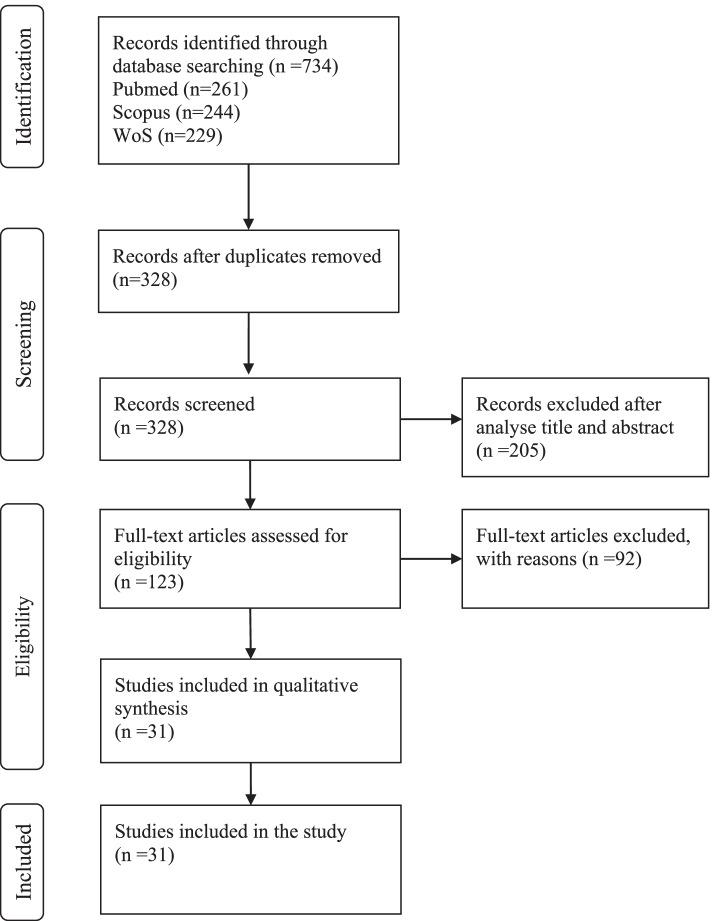
Flow diagram of study selection

From the database search, 734 records were identified. After removing duplicates, the titles and abstracts of 328 articles were screened concerning the eligibility criteria, and 205 were excluded. The full texts of the remaining 1237 articles were evaluated and 92 were excluded for the following reasons: sample characteristics (*n* = 24), data were not analysed regarding the association between PA and mental health variables (*n* = 23), review studies (*n* = 4), no valid instruments to assess PA (*n* = 31) and mental health (*n* = 6), the study was not in the period of the COVID-19 pandemic (*n* = 4). Thirty-one studies were included in this review, 27 about adults and old adults and 4 about children and adolescents. The flow diagram of study search and selection was created according to the Preferred Reporting Items for Systematic Reviews and Meta-Analyses (PRISMA) [23] and is presented in Fig. 1. The mean score of quality was 5.7 ± 1.5. More details are presented in Tables 1 and 2.

**Table 1:** Characteristics and the main results of the association of physical activity/exercise on mental health among adults

Authors	Study design, sample characteristics (n, sex, age), recruitment, country	Outcomes (instruments)	Study quality^**a**^	Main findings
Abate Daga et al. 2021 [24]	Cross-sectional, 595 participants (mean age 30.47 ± 13.57, 50,3% male), online survey between March 9 and April 10, 2020, Italy.	Well-being (WHO-5-J). Physical activity (IPAQ).	**6**	Significant difference in subjective well-being among physical activity rates, inactive people meanly lose 6.53 point of wll-being score versus moderately active and 11,14 points versus very active responders.
Bird et al. 2021 [25]	Cross-sectional, 392 adults (mean age 25.48 ± 5.05; 314 women) recruitment through word-of-mouth and facilitated by social media posts, UK.	Anxiety, depression, and social Dysfunction (GHQ-12). Physical activity (BLPAQ).	6	Planned and unplanned PA were significant explanatory variables for mental health both pre- and during lockdown, but sedentary behaviour was not. Regular PA confers some minor benefits for mental health.
Carvalho et al. 2021 [26]	Cross-sectional, 68 older adults (mean age 74.24 ± 5.67), recruitment by community-based exercise program, Portugal.	Depression (GDS-15). Physical activity (IPAQ-SF).	6	MVPA was significantly higher within the non-depressed group compared with those in the depressed group. Most participants from the depressed group were categorized as low physical activity levels, whereas the majority of the non-depressed group were classified as moderate or high physical activity level.
Carriedo et al. 2020 [27]	Cross-sectional, 483 elderlies (50.9% women, mean age 65.49 ± 5.14), online survey, Spain.	Physical activity (IPAQ). Resilience (CD-RISC). Affects (The Positive and Negative Affect Schedule). Depressive symptoms (six-item self-report scale developed by Kandel and Davies).	4	Older adults who regularly engaged in vigorous (VPA) and moderate-vigorous physical activity (MVPA) during the quarantine reported higher scores in resilience (Locus, Self-efficacy, and Optimism), positive affect, and lower in depressive symptoms.
Cecchini et al. 2021 [28]	Longitudinal, 595 participants (mean age 45.60 ± 15.17, 342 females), four different times during the confinement by telephone call, Spain.	Physical activity (IPAQ). Depressive symptoms (six-item self-report scale developed by Kandel and Davies).	4	In the third week of confidement the risk rate of increasing depressive symptoms affected 68% od the populations, these changes were inversely associated with levels of PA. The 150 min/week of MVPA produced a significant effect in the reduction of depressive symptoms, and the effects can be increased up to 18 h of weekly MVPA. At leas 4 h of MVPA reduce by 49% odds of depressive symptoms.
Chouchou et al. 2020 [29]	Cross-sectional, 400 participants (58.2% women, mean age 29.8 ± 11.5 years), recruitment by online survey between the 35th and 54th days of lockdown, Reunion Island.	Subjective well-being (WHO-5). Sleep quality (PSQI). Physical activity (IPAQ).	6	Those reporting the highest decrease in well-being (4th and 3th quartiles) also reported the highest decrease in their total, moderate and intense weekly PA. Impairment in well-being was independently associated with weekly PA.
Coughenour et al. 2020 [30]	Cross-sectional, 194 universities (73% women, mean age 25.1 ± 7.8 years), online survey between May 7 and May 28, 2020, United States (Southern Nevada).	Estimated cardiorespiratory fitness (algorithm include age, body composition, resting heart rate and PA). Depressive symptoms (PHQ-9).	3	Significant but small correlation between the change in weekly physical activity minutes and the change in PHQ-9 scores.
Eric et al. (2020) [31]	Cross-sectional, 1800 adults (42.7% women, 50.7% aged between 21 and 35 years) online survey, Nigeria.	Subjective well-being (WHO-5). Physical activity (EPQ).	5	Total Daily Energy Expenditure on exercise during the pandemic was found to be significantly related to mental wellbeing.
Faulkner et al. 2020 [32]	Cross-sectional, 8425 participants (70.7% female; mean age 44.5 ± 14.8 years), online survey within the first 2–6 weeks of government-mandated COVID-19 restrictions, UK, Ireland, New Zealand and Australia.	Physical activity (IPAQ). Exercise behaviour change (Stages of Change scale). Subjective well-being (WHO-5). Depressive, anxiety and stress symptoms (DASS-9).	7	Moderate positive correlations between PA and WHO-5 scores and negative correlations between PA and depressive, anxiety and stress symptoms during the initial COVID-19 restrictions.
Glerc et al. 2021 [33]	Cross-sectional, 417 participants (mean age 32.2 ± 13.6, 86.8% women) recruited online through social media between March 24 and May 8, 2020, Canada.	Physical activity (IPAQ). Mental health (PHQ-9). Anxiety (GAD-7). Satisfaction with Life (SWLS).	7	Changes in;VPA accounted for significant variability in the PHQ-9, GAD-7 and SWLS. Participants sufficiently active during covid-19 reported significantly lowe depression and anxiety, and higher life satisfaction.
Lage et al. 2021 [34]	Cross-sectional, 1123 older adults (mena age 67.68 ± 5.91, 91% female), interviewed by telephone call, Brazil.	Depression (GDS-15). Physical activity (IPAQ-SF).		Greater times spent in MPA and MVPA was associated with lower depressive score. Daily walking and sitting time were not associated with depression.
Lesser and Nienhuis, (2020) [35]	Cross-sectional, 1098 participants (79.3% women, mean age 42 ± 15 years) online survey during April and early May 2020, Canada	Physical activity (GLQ). Motivation to exercise (BREQ-3). Anxiety (GAD-7). Overall well-being (MHC-SF).	4	Inactive participants scored significantly lower on the mental health continuum than active participants, though was a non-significant difference in generalized anxiety.
Lin et al. (2020) [36]	Cross-sectional, 628 healthy college students (64.8% female, mean aged 20.18 ± 1.8 years), online survey, China.	Depression (CES-D). Physical activity (IPAQ-SF).	6	Depression negatively correlated with MET-minutes/week in moderate-intensity PA but not vigorous and walking scores.
Marachi et al. 2021 [37]	Cross-sectional, 1669 participants (36.9% aged between 18 and 29 years, 82.4& woman), online survey from April 23 to June 30, 2020, Canada.	Physical activity (PASB-Q) Anxiety (GAD-7) Depression (PHQ-9) Stress (PSS)	3	Participants whose mental health got worse or much worse had greater reductions in physical activity since COVID-19. Mental outcomes such as anxiety relief was the most important motivator to stay active.
Maugeri et al. (2020) [38]	Cross-sectional, 2524 participants (56.4% women, 46% aged between 21 and 40 years), online survey from April 1 to April 30, 2020, Italy.	Physical activity (IPAQ-SF). Well-being (PGWBI).	5	A significant positive correlation was found between the variation of physical activity and mental well-being.
Méndez-Giménez et al. (2020) [39]	Cross-sectional, 4811 participants (61.4% women, 50.7% aged between 27 and 53 years), online survey from March 19 to April 18, 2020, Spain.	Physical activity (IPAQ). Depressive symptoms (6-item self-report scale).	7	PA components were inversely associated with NDS. Performing at least 477 METs-min/week was associated with a 33% decrease in the probability of NDS, and reaching 3000 METs-min/week was associated with the lowest risk of NDS (47%).
Nie et al. 2021 [40]	Cross-sectional, 14,715 participants (aged between 17 and 69 years, 53% male) data collected by online survey between June 20 and July 30, 2020, China.	Physical activity (IPAQ). Mental Health (50-item Self-evaluation table for Chinese residents’mental health).	3	There was a significant association between PA and mental health, the largest associations were seen for home-based group entertainment exercise. Moderate-intensity exercise was better than both light and vigorous intensity exercise for mental health.
Nienhuis and Lesser (2020) [41]	Cross-sectional, 1098 participants (79.3% women, mean age 42 ± 15 years) online survey during April and early May 2020, Canada	Physical activity (GLQ). Motivation to exercise (BREQ-3). Anxiety (GAD-7). Overall well-being (MHC-SF).	4	Women with severe anxiety reported more physical activity those with moderate anxiety. Women’s physical activity levels were more significantly impacted by the increased difficulty and challenge due to the onset of COVID-19 restrictions.
O’brien et al. 2021 [42]	Cross-sectional, 4007 participants (mean age 46.5 ± 14.7 years, 70% female) online survey during 10–26 April 2020, New Zealand.	Physical activity (IPAQ-SF) Depression, Anxiety and Stress (DASS-9). Subjective well-being (WHO-5)	6	Participants who had moderate levels of PA had better mental health status. PA had a strong effect on wellbeing.
Ozdemir et al. (2020) [43]	Cross-sectional, 2301 participants (61.1% women, mean age 36.2 ± 10.9), online survey started 8 weeks after the first case of COVID-19 was officially reported, Turkey.	Physical activity (IPAQ). Depression (BDI). Anxiety symptoms (BAI). Quality of life (WHOQOL- BREF TR).	8	Weak positive relationship between physical activity levels and quality of life, while there was a weak negative relationship between physical activity levels, depression and anxiety.
Savage et al. (2020) [44]	Longitudinal cohort study, 214 students (72% women, mean age 20 years), online survey on the first week of ‘lockdown’ 20 March 2020 and during the fifth week of lockdown 27 April 2020, UK.	Physical activity (EVS). Mental well-being (WEMWBS).	2	Positive association was found between perceived stress and sedentary behaviour.
Stanton et al. (2020) [45]	Cross-sectional, 1491 adults (67% women, mean age 50.5 ± 14.9 years), online survey during April 2020, Australia.	Physical activity (AAS). Depressive and anxiety symptoms (DASS21).	6	Negative changes in physical activity were associated with higher depressive and anxiety symptoms.
Suzuki, et al. 2020 [46]	Longitudinal study, 165 participants (69.7% women, mean age 78.5 ± 8.0 years), mailing questionnaire two moments, one four weeks before the declaration of the state of emergency (from 20 March–15 April), and the second was in the 4 weeks after the declaration of the state of emergency for follow-up (from 16 April to 13 May), China.	Physical activity (PAQ-EJ). Neighbourhood Physical activity (IPAQ-E). Functional health (TMIG-IC). Well-Being (WHO-5-J). Health-Related Quality of Life (SF-12v2).	5	SWB scores significantly decreased in the less active group but this was not seen in the more or equally active group. HRQoL scores were reduced by COVID-19 restrictions regardless of changes in PA.
Trabelsi et al. 2021 [47]	Cross-sectional, 517 older adults (76% young old 56–65 years, 52.2% female) 41 research institutions from Europe, Western-Asia, North-Africa and the American promoted the survey on April 2020.	Well-being (Short Warwick–Edinburgh Mental Wellbeing Scale). Sleep Quality (PSQI) Physical activity (IPAQ)	6	Change in total physical activity energy expenditure was significant predictor of the decrease in mental wellbeing from pre to during lockdown.
Xiang et al. (2020) [48]	Cross-sectional, 1396 college students (36.9% women, mean age 20.68 ± 1.84), online survey, China.	Anxiety and depressive symptoms (SAS and SDS). Physical activity (IPAQ).	6	A high level of physical activity was significantly closely associated with low anxiety, while a moderate or high level of physical activity was significantly associated with reduced depression after adjusting confounding demographic factors.
Zalewska et al. 2021 [49]	Cross-sectional, 141 physiotherapy students (104 women, aged 18–25 years), online survey, Poland.	Depression (Beck Depression Inventory). Physical activity (IPAQ).	3	More physical activity had a positive effect on mental health.
Zhang et al. (2020) [50]	Longitudinal survey, 66 participants (62.12% women, mean age 20.70 ± 2.11), online survey February 19, on March 5 and on March 20, China.	Physical activity (IPAQ). Depressive, anxiety and stress symptoms (DASS21).	3	Physical activity directly alleviated general negative emotions and the maximal mitigation effect occurred when weekly physical activity was about 2500 METs.
Young et al. 2021 [51]	Longitudinal, 20,000 adults (60% women, 93% over the age of 50), recruited from the U.S. Kaiser Permanente Research Bank, online srvey at 3 moments, United States.	Physical activity (Godin Leisure-Time Exercise Questionnaire. Depressive symptoms ((PHQ-2).	5	Participants in the lowest physical activity category (no physical activity) had the highest depression and anxiety scores compared to each successive physical activity category
Carriedo et al. (2020) [27]	Cross-sectional study, 483 elderlies (50.9% women, mean age 65.49 ± 5.14), online survey, Spain.	Physical activity (IPAQ). Resilience (CD-RISC). Affects (The Positive and Negative Affect Schedule). Depressive symptoms (six-item self-report scale developed by Kandel and Davies).	4	Older adults who regularly engaged in vigorous (VPA) and moderate-vigorous physical activity (MVPA) during the quarantine reported higher scores in resilience (Locus, Self-efficacy, and Optimism), positive affect, and lower in depressive symptoms.
Suzuki, et al. (2020) [46]	Longitudinal study, 165 participants (69.7% women, mean age 78.5 ± 8.0 years), mailing questionnaire two moments, one four weeks before the declaration of the state of emergency (from 20 March–15 April), and the second was in the 4 weeks after the declaration of the state of emergency for follow-up (from 16 April to 13 May), China.	Physical activity (PAQ-EJ). Neighbourhood Physical activity (IPAQ-E). Functional health (TMIG-IC). Well-Being (WHO-5-J). Health-Related Quality of Life (SF-12v2).	5	SWB scores significantly decreased in the less active group but this was not seen in the more or equally active group. HRQoL scores were reduced by COVID-19 restrictions regardless of changes in PA.

Abbreviation: *AAS* Active Australia Survey, *BAI* Beck Anxiety Inventory, *BDI* Beck Depression Inventory, *BLPAQ* Brunel Lifestyle Physical Activity Questionnaire, *BREQ-3* Behavioural Regulations in Exercise Questionnaire, *CES-D* Center for Epidemiological Studies Depression Scales, *DASS 21* 21-item Depression, Anxiety and Stress Scale, *DASS-9* Depression Anxiety and Stress Scale-9, *EPQ* Exercise Participation Questionnaire, *EVS* Exercise Vital Sign, *GAD-7* General Anxiety Disorder-7, *GDS-15* Geriatric Depression Scale – 15 items, *GLQ* Godin Leisure Questionnaire, *GHQ-12* General Health Questionnaire-12, *IPAQ* International Physical Activity Questionnaire, *IPAQ-SF* International Physical Activity Questionnaire - Short Form, *MHC-SF* Mental Health Continuum, *MVPA* moderate vigorous physical activity, *NDS* notable depressive symptoms, *PA* physical activity, *PASB-Q* Physical Activity and Sedentary Behavior Questionnaire, *PGWBI* Psychological General Well Being Index, *PHQ-2* 2 item Patient Health Questionnaire, *PHQ-9* 9 item Patient Health Questionnaire, *PSQI* 6-items of the Pittsburgh Sleep Quality Index, *PSS* Perceived Stress Scale, *SAS* Self-Rating Anxiety Scale, *SDS* Self-Rating Depression Scale, *WEMWBS* Warwick-Edinburgh Mental Wellbeing Scale, *WHO-5* 5-World Health Organization Well-Being index, *WHOQOL- BREF TR* World Health Organization Quality of Life Scale, *VPA* Vigorous Physical Activity, *MVPA* Moderate-vigorous Physical Activity, *CD-RISC* The Connor-Davidson resilience scale, *SWB* Subjective Well-Being, *TMIG-IC* Tokyo Metropolitan Institute of Gerontology Index of Competence, *IPAQ-E* International Physical Activity Questionnaire Environment Module, *PAQ-EJ* Physical Activity Questionnaire for Elderly Japanese, *WHO-5-J* World Health Organization’s Five Well-being Index, *SWLS* Satisfaction with Life Scale, *SF-12v2* Medical Outcome Study 12-Item Short-Form Survey v2, *SWB* Subjective Well-Being

^a^According to the Newcastle-Ottawa Scale (NOS)

**Table 2:** Characteristics and the main results of the association of physical activity/exercise on mental health among children and adolescents

Authors	Study design, sample characteristics (n, sex, age), recruitment, country	Outcomes (instruments)	Study quality^**a**^	Main findings
Alves, et al. (2020) [52]	Longitudinal study, 64 children (63% girls, mean age 11.84 ± 1.28 years), phone or video call visit one from April 22nd to June 26th, visit two from May 22nd to July 29th, 2020, USA.	Physical Activity (PAR). Anxiety (STAIC). Positive and Negative Affect (PANAS-C).	5	MVPA was associated with less state anxiety, sedentary time, leisure screen time and VPA was not associated with lower state anxiety. Negative affect was correlated with sedentary time and leisure screen time. Positive affect was not related to any of the physical activity measures.
Chi, et al. (2020) [53]	Cross-sectional study, 1794 adolescents (43.8% girls, mean age 15.2 ± 0.4 years), online survey between May 13 and 20, 2020, shortly after reopening schools, China.	COVID-fear (FCV-19S). Nutrition (HPLP-II). Physical Activity (IPAQ-SF). Insomnia (YSIS). Depressive symptoms (PHQ-9). Anxiety (GAD-7).	8	With lowly active physically as the referent, moderately physically was significantly associated with a lower level of depressive symptoms and anxiety symptoms while highly active physically was associated with a lower level of insomnia symptoms, depressive symptoms and anxiety symptoms.
Morres, et al. 2021 [54]	Cross-sectional, 950 adolescents (mean age 14.41 ± 1.63 years, 518 boys), web-based survey, Greece.	Physical activity (IPAQ-SF) Mood (4DMS) Psychological well-being (WHO-5)	5	Increased physical activity predicted better well-being. Days of PA per week was stronger predictor of well-being than minutes of PA per week. Both in-house and out-of-house PA were beneficial.
Kang, et al. (2020) [55]	Cross-sectional study, 4898 adolescents (52% girls, mean age 16.3 ± 1.3 years), online survey between March 8th and 15th, 2020, China.	Physical Activity (IPAQ). Mood states (Profile of Mood States).	6	Higher levels of PA were significantly associated with lower levels of total mood disturbance.

Abbreviation: *PA* physical activity, *PAR* 24-h physical activity recall, *STAIC* State-Trait Anxiety Inventory for Children, *PANAS-C* Positive and Negative Affect Schedule for Children, *MVPA* Moderate and vigorous physical activity, *VPA* Vigorous Physical Activity, *FCV-19* Chinese version of the Fear of COVID-19 Scale, *HPLP-II* sub-scale of the Chinese version of the Health Promoting Lifestyle Profile-II, *IPAQ-SF* International Physical Activity Questionnaire Short Form, *YSIS* Youth Self-Rating Insomnia Scales, *PHQ-9* 9-item Patient Health Questionnaire, *GAD-7* Generalized Anxiety Disorder scale, *PA* Physical Activity, *IPAQ* International Physical Activity Questionnaire, *WHO-5* World Health Organization’s Five Well-being Index, *4DMS* 4-Dimensional Mood Scale

^a^According to the Newcastle-Ottawa Scale (NOS)

### The association between physical activity and mental health among adults and old adults

The details of the association between PA and mental health among adults and old adults are summarised in Table 1, 27 studies were included [24–49, 51].

#### Participants characteristics and date of filling the questionnaires

The number of participants in the 27 included studies varied between 66 [50] and 14,715 [40] participants. Regarding sex, with exception of four studies [24, 31, 40, 48] the majority included more women than men. Concerning age, most articles presented the mean age range between 20 and 30 years [24, 25, 36, 37, 44, 48–50, 56, 57], two article presented mean age between 30 and 40 years [33, 43]; five articles presented mean age between 40 and 50 years [28, 32, 35, 41, 42]; and seven articles presented mean age above 50 years [26, 27, 34, 45–47, 51]. Four articles did not present mean age [31, 38–40], for one article the age ranged between 21 and 35 years [31], for another, the age ranged between 21 and 40 years [38], for another, the age ranged between 17 and 69 years [40], and in one the age ranged between 27 and 53 years [39]. The majority of studies reported, the sample filled out online questionnaires. One study used interviewed by telephone call [34]. Twenty four studies conducted a cross-sectional analysis and four a longitudinal analysis.

#### Study location

The studies were carried out on five different continents. Ten studies from Europe [24–28, 38, 39, 43, 44, 49], foure studies from Asia [36, 40, 46, 48, 50], seven studies from America [33–35, 37, 41, 51, 57] two studies from Africa [31, 56], two study from Oceania [42, 45], and two multi-centre study [32, 47].

#### Outcomes and instruments

Concerning outcomes and instruments, 12 articles used the International Physical Activity Questionnaire (IPAQ) to assess PA and one article calculated an estimate of cardiorespiratory fitness (algorithm includes age, body composition, resting heart rate and PA) [57]. The others articles assessed PA with different instruments. Mental health included analyses of subjective well-being, sleep quality, depressive symptoms, anxiety, quality of life, psychological distress, motivation, resilience, affects (positive and negative), and health-related quality of life.

#### Main findings

Overall, all articles found a positive association between PA and better outcomes of mental health (e. g., depression, anxiety, well-being). Physical activity was explanatory variable for mental health [25]. Physical activity was positive associated with mental health [42, 49]. Articles that observed a decrease in PA during the stay-at-home order also observed a decrease in well-being [47, 56], negative changes in depressive symptoms [57], and negative changes in anxiety and stress symptoms [45]. This relationship seems to be bidirectional, since participants who decrease in mental health had greater reduction in physical activity [37]. Inactive people had worse well-being, highest depression and enxiety compared with moderately active and very active participants [24, 51]. Also, inactive old adults had more depressive symptoms [26]. On the other hand, participants sufficiently active reported significantly lower depression and anxiety and higher life satisfaction. Moreover, it was found that exercise intensity seems to be important. Two studies founded that depression was significantly negatively correlated with moderate-intensity PA but not vigorous and walking/light exeeercuse [36, 40]. Another one found that vigorous PA better predicted depressive symptoms than moderate PA [27]. Three studies suggested that the threshold of PA should be done to fells the benefits on mental health [28, 39, 50]. At least 4 h of MVPA reduced by 49% odds of depressive symptoms [28], and at leas 477 METs-min/week was associated with a 33% decrease in the probability of depressive symptoms [39]. On the other hand, a non-significant association was found between PA and anxiety [35], and between PA and health-related quality of life [46]. One study found that the decrease in mental wellbeing and increase in perceived stress was not related to changes in PA [44].

### The association between physical activity and mental health among children and adolescents

The details of the association between PA and mental health among children and adolescents are shown in Table 2, four studies were included [52–55].

#### Participants characteristics and date of filling the questionnaires

The number of participants ranged between 64 and 4898 children and adolescents. More girls than boys participated in the studies. The mean ages were 11, 14, 15, and 16 years old. One study was longitudinal and presents two moments of assessment, also opting for phone or video calls to collect the data [52]. The other three studies were cross-sectional, and collected data by online surveys.

#### Study location

One study was from the USA [52], one from Greece [54] and the other two were from China [53, 55].

#### Outcomes and instruments

Three studies assessed PA by the International physical activity questionnaire (IPAQ) questionnaire [53–55], and another one used a 24-h physical activity recall [52]. Regarding mental health, different outcomes were assessed, such as anxiety, positive and negative affect, insomnia, depressive symptoms, psychological well-being and mood states.

#### Main findings

Moderate physical activity was associated with less state anxiety [52, 53]. Positive affect was not related to physical activity [52]. Higher levels of physical activity were also significantly associated with lower levels of total mood disturbance [55]. Regarding the dose of physical activity, days of physical activity per week was stronger predictor of well-being than minutes of physical activity per week [54].

## Discussion

This systematic review focuses on the association between PA and mental health during the first year of the COVID-19 pandemic. In particular, we sought to answer if PA mitigates the effects of the stay-at-home order on mental health. The COVID-19 pandemic generated numerous challenges for public health, particularly the significant burden of mental health in the population [58, 59]. In addition, PA has been recognized as an effective mitigation strategy for improving mental health [60]. The COVID-19 pandemic has been affecting all continents in the world, at different scales. This study analysed 31 research articles, 27 about adults and old adults and 4 about children and adolescents. The articles are mainly based on cross-sectional studies and five are longitudinal studies. In nearly all of the studies comprised in the present systematic review, investigators used online surveys as the main procedure to collect data. Overall, the studies suggested that higher PA is associated with less negative mental health symptoms, such as depression, anxiety, well-being, and fear, independently of age.

The studies observed that women showed more depressive symptoms than men [28, 39, 50, 53, 55], and this trend increases with age [24] Furthermore, individuals with a lower level of masculinity traits (not specifically females) increased risk of developing depression [36], and women experienced more generalised anxiety [41]. The reduction of PA levels may mostly influence the mental well-being of females [38, 49]. Those findings are expected since the literature is consistent in signalising sex differences in most mental disorders [61]. On the whole, the prevalence rates of anxiety and depression were both higher than the rates found in previous studies before the COVID-19 pandemic [36, 37, 43, 45, 48, 57], which highlights a worsening in mental health during the first year of the COVID-19 pandemic. Regarding age, younger individuals experienced significantly higher anxiety and depression, also income influenced mental health, lower-income participants present worse mental health [37].

Five articles conducted a longitudinal study. Among those studies, four enrolled adults and old adults [28, 44, 46, 50, 51] and in one the sample comprised children [52]. Among the studies that have collected measures before and after the stay-at-home order, both observed a significant reduction in PA [44, 46]. Two study collected measures after the stay-at-home order and during this period physical activity mean score decreased minimally [51], and depressive symptoms increase as the weeks of isolation go [28]. Other studies also reported a reduction in self-reported PA [26, 33, 34, 37, 38, 42, 43, 45, 56, 57] and increase in sedentary behaviour [25, 26, 34]. Individuals who reported larger decrease in MVPA pre to during COVID-19 reported relatively poorer mental health [33]. Participants whose mental health got “worse” or “much worse” had greater reductions in physical activity [37]. Also, increased levels of physical activity were associated with stronger effects on wellbeing [42]. The reduction was more pronounced in men than women [32, 38, 44], in vigorous PA [38], and between those with lower health-related quality of life scores before the COVID-19 pandemic [46]. The possible explanation for this sex-difference is that men are more engaged in group/community PA and sports in clubs or gyms, and those were more impacted by the COVID-19 restrictions. Also, women are more engaged in low and moderate physical activities, which can be done at home. Besides, women spent more time in housework activities. Women without changes in childcare provision reported more opportunities to be physically active [41]. The same sex-differences were observed in an Italian study [62]. Increases in PA were observed for a minority, but the majority of the respective study samples demonstrating a positive change were individuals who did not meet recommended PA guidelines before the COVID-19 pandemic [32, 38]. Additional reasons could be an increase in awareness for health issues and more time to pursue PA during the stay-at-home order [32]. These behaviour changes can help to maintain a more active lifestyle during the pandemic. Another study found an increase of 40% in PA in a sample that was already active before the COVID-19 pandemic [35]. PA could be used as a coping strategy to deal with the consequences of the pandemic. The place where individuals prefer to practice PA seems to be important, since active participants reported greater connectedness to nature and nature relatedness than the inactive population [35].

There was no consensus across studies for the optimal PA levels for mitigating negative mental symptoms. The more the physical activity is frequent and vigorous, the best people feel themselves [24]. Among Chinese students, 2500 METs minute/week of PA every week was the optimal dose to alleviate negative emotion [50]. On the other hand, a Spanish community sample study showed that 477 METs-minute/week was associated with a 33% decrease in the probabilities of notable depressive symptoms [39]. The difference between the values must be relativised considering the samples’ characteristics. The first one concerns students with a mean age of 20 years [50], and the second one concerns a community sample with a mean age of 43.2 years for women and 40.5 years for men [39]. In addition, it is claimed that at least 3000 METs-minute/week reduce the odds of depressive symptoms by 47% [39]. These studies used the IPAQ to assess PA, and according to IPAQ, to reach a minimally active category at least 600 METs minute/week is needed [63]. The American College of Sports Medicine also recommends for healthy adults aged 18–65 years at least 600 METs minute/week but did not specify the minimum dose to prevent depressive symptoms [14, 64].

Studies also examined the association between PA and mental health according to PA intensity. Moderate-intensity PA (e.g., walking or jogging on a treadmill, using an elliptical trainer, cleaning house) is associated with better mental health outcomes than vigorous-intensity PA [36, 40], and light-intensity PA [40]. On the other hand, vigorous-intensity PA better predicted depressive symptoms than moderate-intensity PA; also the effect size was higher for the association between vigorous-intensity PA and level of resilience compared with moderate-intensity PA [27]. One study found that performing high PA levels has no positive effect on depressive symptoms [39]. Another study explored the type of PA and showed that stretching and resistance training were associated with lower anxiety, and three types of PA (household chores, stretching, and resistance training) were associated with lower depression symptoms [48].

One study explored the association of specific types of physical exercise and mental health, and founded that home-based group entertainment exercise, rope skipping and badminton, Chinese traditional sports, video dancing and sensory-motor games present a greater reduction in mental health than others types [40].

Sedentary behaviour was observed in few studies and contradictory findings were observed. No association between sedentary time and depressive symptoms was observed [26, 36]. However, other studies have shown that sedentary behaviour was associated with poorer mental health [25], well-being [32] and perceived stress [44].

Concerning the association between PA and mental health outcomes among children and adolescents, only four articles were selected. Some issues must be highlighted. This population had to face, beyond the reality that changed from the COVID-19 pandemic, the changes in the education system such as online learning became the main learning method for students and uncertainty of academic development, which probably caused more anxiety level [52, 53]. Both moderate and highly active groups were significantly associated with less depressive symptoms [53] and anxiety [52, 53], and only the most active adolescents reported significantly lower insomnia symptoms [53] and better mood states [55]. Days of physical activity per week was stronger predictor of well-being than minutes of physical activity per week [54].

Regarding old-age samples, the studies mentioned the particular vulnerability to changes in social circumstances, and highlight the importance of health-related quality of life [46] and levels of resilience [27] to deal with the consequences of the COVID-19 pandemic on the PA level. The stay-at-home order can cause greater distress and feelings of sadness, considered specific risk factor for depressive symptoms [34]. Mental health and physical activity decrease pre to during stay-at-home order [47]. In a group that were previously regular participants of a formal exercise program, MVPA was significantly higher within the non-depressed group compared with depressed group [26]. Being active previously of COVID-19 confinement did not prevent 30.4% of Brazilian older adults from having depressive symptoms, but these results is much lower than prevalence of depression in Brazilian general population, which is 68% [34].

The present systematic review had some limitations that must be mentioned. First, the studies present correlative analyses, not causal ones, thus randomised controlled trials must be conducted in the context of the COVID-19 pandemic and the stay-at-home order to clarify the direction of the association. However, beyond the COVID-19 context, randomised controlled trials showed that PA interventions show beneficial effects on mental health outcomes such as depression and anxiety [65]. Thus, a nuanced perspective particularly during the COVID-19 context in future research is needed. Moreover, the included studies with community samples were limited, and the analyses were mostly based on convenience samples with college students, which had specific characteristics and low mean age. Thus, future research needs to focus on representative study samples.

## Conclusion

This review helps to clarify the positive association between PA and mental health during the first year of the COVID-19 pandemic, especially considering the effects of the stay-at-home order worldwide. Although there is an association between increased PA and improved mental health, further studies are needed, specifically randomised clinical trials, to identify the direction of this relationship, and what kind of PA, intensity, and frequency are most indicated to maximise the effects. Also, an investigation to examine the association during the second year of the COVID-19 pandemic is needed. The impact of the COVID-19 pandemic on mental health may be continuous and long-term [66, 67]. Thus, public health agencies must provide timely and effective interventions, in which PA and exercise should be a priority.

## Data Availability

Data sharing is not applicable to this article as no datasets were generated or analysed during the current study.

## Abbreviations

COVID-19: Coronavirus disease-19
SARS-CoV-2: Severe acute respiratory syndrome coronavirus-2
PA: Physical activity
WHO: World Health Organization
IPAQ: International physical activity questionnaire
MET: Metabolic equivalent
